# A Readout Mechanism for Latency Codes

**DOI:** 10.3389/fncom.2016.00107

**Published:** 2016-10-20

**Authors:** Oran Zohar, Maoz Shamir

**Affiliations:** ^1^Department of Brain and Cognitive Sciences, Ben-Gurion University of the NegevBeer-Sheva, Israel; ^2^Zlotowski Center for Neuroscience, Ben-Gurion University of the NegevBeer-Sheva, Israel; ^3^Department of Physiology and Cell Biology, Ben-Gurion University of the NegevBeer-Sheva, Israel; ^4^Department of Physics, Ben-Gurion University of the NegevBeer-Sheva, Israel

**Keywords:** spike latency, winner takes all, fast readout, temporal code, conductance based model, rate model

## Abstract

Response latency has been suggested as a possible source of information in the central nervous system when fast decisions are required. The accuracy of latency codes was studied in the past using a simplified readout algorithm termed the temporal-winner-take-all (tWTA). The tWTA is a competitive readout algorithm in which populations of neurons with a similar decision preference compete, and the algorithm selects according to the preference of the population that reaches the decision threshold first. It has been shown that this algorithm can account for accurate decisions among a small number of alternatives during short biologically relevant time periods. However, one of the major points of criticism of latency codes has been that it is unclear how can such a readout be implemented by the central nervous system. Here we show that the solution to this long standing puzzle may be rather simple. We suggest a mechanism that is based on reciprocal inhibition architecture, similar to that of the conventional winner-take-all, and show that under a wide range of parameters this mechanism is sufficient to implement the tWTA algorithm. This is done by first analyzing a rate toy model, and demonstrating its ability to discriminate short latency differences between its inputs. We then study the sensitivity of this mechanism to fine-tuning of its initial conditions, and show that it is robust to wide range of noise levels in the initial conditions. These results are then generalized to a Hodgkin-Huxley type of neuron model, using numerical simulations. Latency codes have been criticized for requiring a reliable stimulus-onset detection mechanism as a reference for measuring latency. Here we show that this frequent assumption does not hold, and that, an additional onset estimator is not needed to trigger this simple tWTA mechanism.

## Introduction

Neuronal response latency has been shown to be tuned to various features of external stimuli across many sensory modalities including the somatosensory (Panzeri et al., 2001; Johansson and Birznieks, 2004; Panzeri and Diamond, 2010), auditory (Brugge et al., 1996, 2001; Klug et al., 2000; Leibold and van Hemmen, 2005; McAlpine, 2005; Zhou et al., 2005; Chase and Young, 2007; Joris and Yin, 2007; Goodman and Brette, 2010; Grothe et al., 2010; Ashida and Carr, 2011; Grothe and Koch, 2011; Lüling et al., 2011; Zohar et al., 2011; Shamir, 2014), and visual systems (Hubel and Wiesel, 1968; Gawne et al., 1996; Van Rullen and Thorpe, 2001; Gollisch and Meister, 2008; Shriki et al., 2012; Shamir, 2014). In addition, latency tuning has been observed in various stations along the information processing pathway from the receptor level (Hall et al., 1995; Van Rullen and Thorpe, 2001; Gollisch and Meister, 2008; McGinley et al., 2012), the brainstem (Klug et al., 2000; Joris and Yin, 2007; Grothe and Koch, 2011; Zohar et al., 2011) and up to the cerebral cortex (Hubel and Wiesel, 1968; Brugge et al., 1996, 2001; Shriki et al., 2012). For this reason, response latency has been suggested as a possible source of information in cases where fast decisions are required (Gawne et al., 1996; Gautrais and Thorpe, 1998; Van Rullen and Thorpe, 2001; Johansson and Birznieks, 2004; Chase and Young, 2007; Gollisch and Meister, 2008; Shamir, 2009, 2014; Goodman and Brette, 2010; Panzeri and Diamond, 2010; Zohar et al., 2011; Shriki et al., 2012).

The accuracy of latency codes has been investigated using the framework of a simple latency based competitive readout, the “*temporal-winner-take-all”* (tWTA), in a modeling study (Shamir, 2009) and more recently in the auditory (Zohar et al., 2011) and the visual systems (Shriki et al., 2012). The tWTA estimates the stimulus on the basis of the preferred stimulus of the neuron (or population) that fired first (or reached a certain decision threshold). The utility of the tWTA is that it is sufficiently simple and well-defined to enable analytical investigation of readout speed and accuracy. It was shown that the tWTA can account for fast and accurate discrimination between a small number of alternatives. However, it remains unclear how the central nervous system implements such a readout, if at all. This raises crucial questions as to the utility of latency coding in the brain.

The conventional rate winner-take-all (WTA) readout that estimates the stimulus based on the preferred stimulus of the neuron that fired the most spikes (rather than the first as in tWTA) has been widely used and studied in neuroscience (Fukai and Tanaka, 1997; White et al., 1998; Jin and Seung, 2002; Laing and Chow, 2002). There is a general consensus that the computation of the WTA decision is based on reciprocal inhibition between groups of neurons with similar preferences.

Here we show that the basic WTA architecture is also sensitive to the temporal order of its inputs such that it is able to implement the tWTA readout. This paper is organized as follows. First we define the basic reciprocal inhibition architecture of the tWTA mechanism and analyze the system using the framework of a rate toy model to facilitate the analytical investigation. Then, we study the generalization of our analysis to a model of spiking neurons in a numerical study of conductance-based neurons. Finally, we discuss the issue of estimating the stimulus onset time and the implications of the findings.

## Methods

### Spiking neurons model

In the numerical simulations of spiking neurons we used the following conductance-based model with typical parameters taken from Shriki et al. (2003) and Shamir et al. (2009).

$$\begin{array}{r} C\frac{dV_{i}}{dt}=I_{i}^{leak}+I_{i}^{net}+I_{i}^{ext}+I_{i}^{active} \end{array}$$

where $I_{i}^{leak},I_{i}^{net},I_{i}^{ext},I_{i}^{active}$ denote the leak, reciprocal inhibition, upstream input and active currents, respectively, and *C* = 1μF/cm^2^ is the membrane capacitance. The currents obey $I_{i}^{leak}=g_{L}(E_{L}-V_{i}(t)),I_{i\in \left\{pop_{A}\right\}}^{net}=\frac{2{\text{g}}_{\text{S}}}{\text{N}}\sum_{j\in \left\{pop_{B}\right\}}s_{ij}\left(t\right)\left(E_{s}-V_{i}\right),I_{i}^{active}=I_{Na}+I_{K}+I_{A}$ with $I_{Na}=g_{Na}m_{\infty }^{3}h\left(V-E_{Na}\right)$, $I_{K}=g_{K}n^{4}\left(V-E_{K}\right)$, $I_{A}=g_{A}a_{\infty }^{3}b\left(V-E_{K}\right)$. The parameters *E*_*L*_, *E*_*s*_, *E*_*Na*_, *E*_*K*_ denote the reversal potentials of the ionic currents in m V and are −65, −80, 55, −80^,^ respectively. Conductance is given in units of ms/cm^2^, and *g*_*L*_, *g*_*Na*_, *g*_*K*_, *g*_*A*_ are 0.05, 100, 40, 20^,^ respectively. The value of g_S_ is discussed below. The variables *s*_*ij*_, *m, h, n, a, b* obey the following dynamics;

$$\begin{array}{rcl} ds_{ij}/dt & = & 1+\left(\text{tanh}\left(V_{pre}/10\right)/2\right)\left(1-s\right)/\left(\tau_{R}\right)-s/\tau_{D},db/dt\\ = & \left(b_{\infty }-b\right)/\tau_{b} \end{array}$$

*dx*/*dt* = (*x*_∞_ − *x*)τ_*x*_ for *x* = *n, h, b* with *b*_∞_ = 1/(exp((*V* + 80)/6) + 1), *a*_∞_ = 1/(exp(−(*V* + 50/20)) + 1) and *x*_∞_ = α_*x*_/α_*x*_ + β_*x*_ for *x* = *m*, *h*, *n*.

Time constants are measured in ms, andτ_*b*_, τ_*D*_, τ_*R*_ are 20, 10, 0.5^,^ respectively, and τ_*x*_ = ϕ/α_*x*_ + β_*x*_ for *x* = *m*, *h*, *n*, where ϕ = 0.1 and

$$\begin{array}{l} \beta_{m}=4\;\exp\;\left(-\left(V+55\right)/18\right), \end{array}$$

$$\alpha_{m}=-(0.1(V+30)/(\exp(-0.1(V+30)-1),$$

$$\begin{array}{l} \beta_{h}=1/\left(\exp\;\left(-0.1\left(V+14\right)\right)+1\right), \end{array}$$

$$\begin{array}{l} \alpha_{h}=0.07\;\exp\;\left(-\left(V+44\right)\right), \end{array}$$

$$\begin{array}{l} \alpha_{n}=-\left(0.01\left(V+34\right)\right)/\left(\exp\;\left(-0.1\left(V+34\right)\right)-1\right), \end{array}$$

$$\begin{array}{l} \beta_{n}=0.125\;\exp\;\left(-\left(V+44\right)/80\right). \end{array}$$

$I_{x,i}^{ext}\left(t\right)=r\Theta \left(t-\left[T+\tau_{x}+\delta_{x,i}\right]\right)+\xi_{x,i}\left(t\right)$ is the input current to neuron *i* in population *x* ∈ {1, 2} from the upstream population, where Θ is the Heaviside step function, *T* is an absolute delay in the response and τ_*x*_ is stimulus selective delay (see A with τ_*i*_ = 0 at the preferred stimulus, and τ_*i*_ = τ). The input current contained two sources of noise. One was a random jitter in the input latency to each neuron, denoted by δ_*x, i*_, that were taken to be i.i.d. Gaussian random variables with zero mean and standard deviation Δ, $\delta_{x,i}\sim N\left(0,\Delta^{2}\right)$. The second source was an additive Gaussian white noise term, ξ_*x, i*_(*t*), that was drawn i.i.d with ξ(*t*) ~ *N*(0, Ξ^2^). In the numerical simulations we took *r* = 5mA/cm^2^, and the white noise, ξ_*x, i*_(*t*), was binned at 1ms time intervals with Ξ = 5mA/cm^2^.

## Results

**Figure 1:**
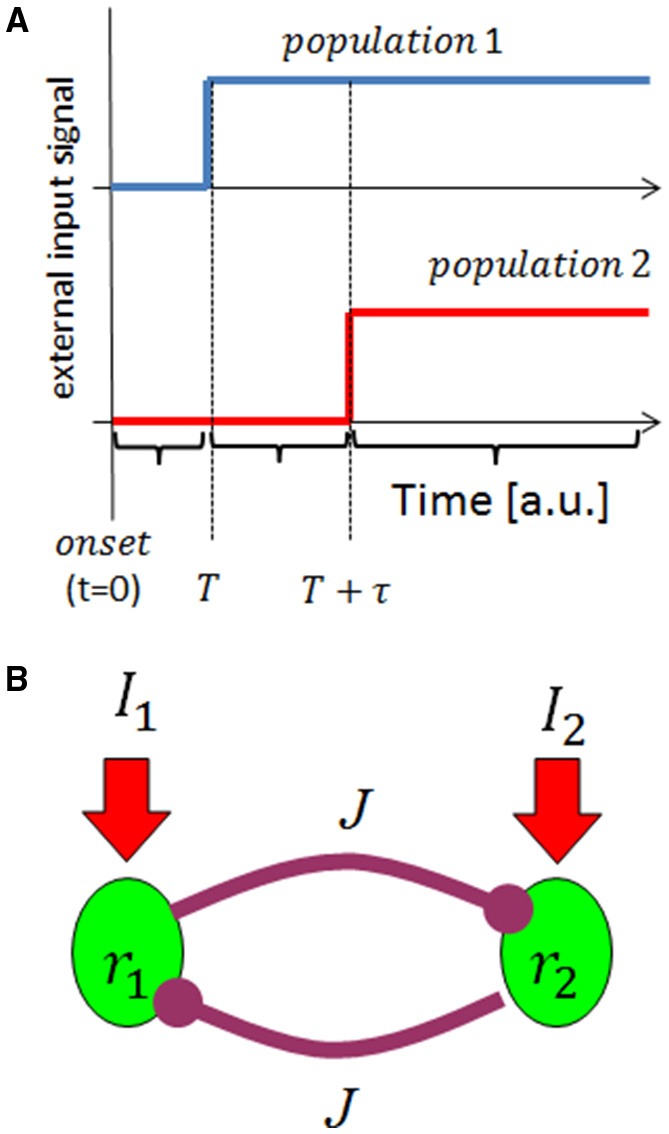
**Schematic illustration of the model**. **(A)** The input model: we consider input from two upstream populations that encode the stimulus identity by their response latency. The traces show the rate of the two populations (in different colors) as a function of time, given the preferred stimulus of population 1 is presented at time *t* = *0*. **(B)** Network architecture. The upstream population's responses serve as input to a reciprocal inhibition WTA type network.

We first studied the tWTA readout mechanism using the framework two-alternative forced choice discrimination in a rate model. The readout mechanism receives two inputs, *I*_1_ and *I*_2_. These inputs represent the activities of two upstream populations of neurons responding to an external stimulus, each of which is characterized by its own preferred stimulus. For example, *I*_1_ can be thought of as the response of a population of inferior colliculus auditory neurons with a preferred sound source azimuth to the right of the animal, whereas *I*_2_ is the response to the left. The response latency of the two inputs is assumed to be tuned to the external stimulus. The task of the readout mechanism is to infer the stimulus based on the inputs it receives. For purposes of studying the ability of the readout to select according to the latency of its inputs, we further assume that both inputs have the same strength; however, the input corresponding to the “correct choice” responds faster in some sense. Specifically, we modeled the inputs as a step function, *I*_*i*_(*t*) = Θ(*t* − *T* − τ_*i*_), where Θ is the Heaviside step function, *T* is an absolute delay in the response and τ_*i*_ is the stimulus selective delay (latency tuning: τ_*i*_ = 0 at the preferred stimulus, and τ_*i*_ = τ > 0 otherwise); Figure 1A.

**Figure 2:**
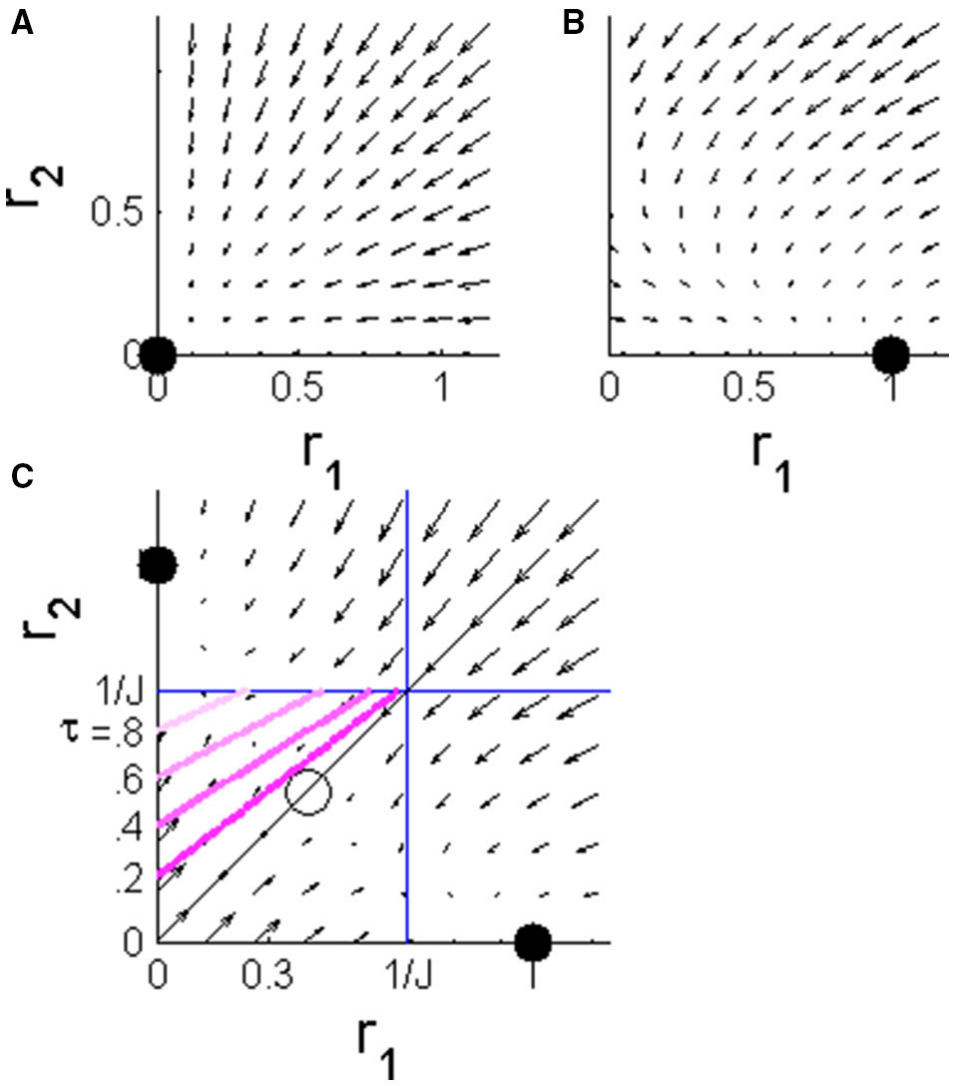
**The phase plane of the WTA rate model during different stages of the dynamics**. The arrows show the vector $\left(\begin{array}{c} {\dot{r}}_{1}\\ {\dot{r}}_{2} \end{array}\right)$ in the plane of [*r*_1_, *r*_2_]. **(A)** Before the input populations begin to respond to the stimulus, *t* < *T*, the system is dominated by a single fixed point at the origin. **(B)** During the stimulus selective response period of the upstream input, *T* < *t* < *T* + τ, the single fixed point shifts to a state in which population 1 outrivals population 2 (in the case of input in the preferred stimulus of population 1, as in the example in Figure 1). **(C)** After the stimulus selective response, *T* + τ < *t*, both inputs to the competing populations are the same. The system has two stable fixed points corresponding to the states in which one population outrivals the other and fully suppresses it. The identity line (solid black) is the borderline between the basins of attraction of the two fixed points. The pink lines show the borderlines between the regions of the phase plane in which the initial conditions will result in a correct decision (below the line) and an incorrect decision (above) for different values of τ = 0.2, 0.4, 0.6, and 0.8; that is, the initial conditions that lead to *r*_2_(*T* + τ) = *r*_1_(*T* + τ).

Our tWTA mechanism is based on reciprocal inhibition between the two populations, Figure 1B. Denoting by *r*_*i*_ the mean firing rate of neurons in the *i*th population (*i* = 1, 2), the neuronal dynamics in our model obey

(1) $${\dot{r}}_{1}=-r_{1}+g(I_{1}-Jr_{2})$$

(2) $${\dot{r}}_{2}=-r_{2}+g(I_{2}-Jr_{1})\;$$

where *J* is the strength of the reciprocal inhibition, and *g*(*x*) is typically modeled by a sigmoidal function. Here, for simplicity of the analysis, we used a threshold-linear function, *g*(*x*) = *x* for *x* > 0 and *g*(*x*) = 0 otherwise.We now consider the dynamics in the case where the stimulus is the preferred stimulus of population 1, consequently population 1 receives the “faster” input. During the absolute delay period (i.e., from stimulus onset at time *t* = 0 to time *T*, see Figure 1A) the downstream populations have yet to respond to the stimulus, *I*_1_ = *I*_2_ = 0. In this case, the system is governed by a single stable fixed point at the origin, *r*_1_ = *r*_2_ = 0, to which the system converges exponentially; Figure 2A. For the duration of the stimulus selective response, *t* ∈ [*T, T* + τ] the single fixed point changes to (1, 0) in which population 1 “outrivals” population 2, in this case; Figure 2B. This fixed point will serve as an attractor and the system will be drawn toward it. At longer times, *t* > (*T* + τ), both populations receive the same level of input from the upstream population. If the reciprocal inhibition is weak, *J* < 1, no population can outrival the other and the system will converge to a symmetric fixed point in which *r*_1_ = *r*_2_. On the other hand, for sufficiently strong inhibition, *J* > 1, the system has two stable fixed points in which one population outrivals the other, Figure 2C. Due to symmetry, the basin of attraction of the two fixed points is separated by the identity line, *r*_1_ = *r*_2_.

In the absence of noise, the initial conditions are expected to be at the origin. Consequently, any amount of stimulus selective delay, τ > 0, will tip the system into the correct basin of attraction. Thus, this trivial architecture produces infinite sensitivity to response latency, in the sense that it achieves correct discrimination for every positive τ.

This hypersensitivity results from bi-stable dynamics where the initial conditions are exactly on the borderline between the basins of attraction of both fixed points. However, neural activity is inherently stochastic and it is unreasonable to assume the WTA type competition will start from such fine-tuned initial conditions. To study the robustness of this readout mechanism we added noise to the initial conditions and investigated the dependence of the tWTA accuracy on the noise level. Thus, instead of starting the WTA competition exactly at the origin, *r*_1_ = *r*_2_ = 0, we now assume that prior to the stimulus selective response period the activity of both populations fluctuates such that the WTA dynamics starts from a “cloud” of distribution close to the origin. Specifically, for the sake of analytical simplicity, we assume the initial conditions *r*_1_(*T*) and *r*_2_(*T*) to be independent and identically distributed exponential random variables with a standard deviation σ, at the onset of the tWTA competition at the beginning of the stimulus selective response, time *t* = *T*. Assuming further that the noise level is small relative to1/*J*, one obtains that for *t* + *T* ∈ [*T, T* + τ]

$$\begin{array}{l} r_{1}(T+t)=1-r_{1}(T)J{\text{e}}^{-t}t+\left(r_{1}(T)-1\right){\text{e}}^{-t}\\ r_{2}(T+t)=r_{2}(T){\text{e}}^{-t}\; \end{array}$$

where we have assumed without loss of generality that stimulus 1 was presented first. During the stimulus selective response the dynamics attracts the system toward the “correct” fixed point, “outrival 1” in this case. An incorrect decision will occurs when the system is at the basin of attraction of “outrival 2” at the end of the stimulus selective period, namely *r*_2_(*T* + τ) > *r*_1_(*T* + τ), which is translated to *r*_2_(*T*) > *ar*_1_(*T*) + *b*, where *a* = 1/(1 + *Jτ*), and *b* = (e^τ^ − 1)/(1 + *Jτ*); see pink lines in Figure 2C. Averaging over the distribution of the initial conditions yields the probability of a correct discrimination, *Pc*, in this approximation

(3) $$\begin{array}{l} P_{C}=1-\frac{{\text{e}}^{-\frac{b}{\sigma }}}{a+1} \end{array}$$

**Figure 3:**
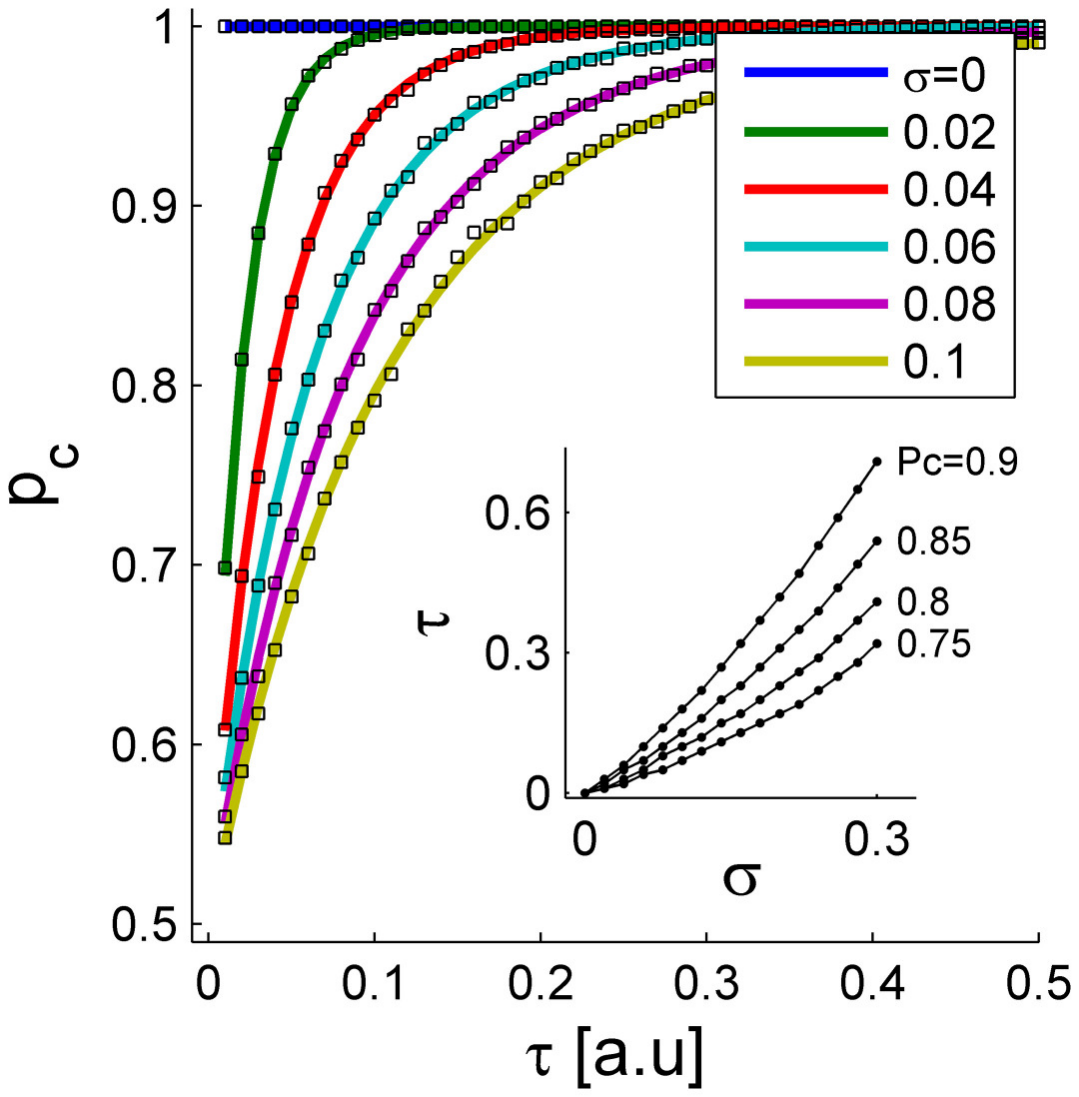
**The probability of correct discrimination is shown as a function of the stimulus selective delay, τ, for different initial conditions of noise levels, σ (different colors)**. The solid lines depict the analytical approximation of Equation (3). The squares show the numerical estimation obtained by averaging the results of the simulation decision over 10^4^ repetitions. The inset shows the “signal”, τ, required to obtain a specific level of performance, P_*C*_, as a function of noise level, σ. Here we used *J* = *1.1*.

Figure 3 shows the probability of correct discrimination as a function of τ for different noise levels in the initial conditions, σ, in different colors. The solid lines show the analytical approximation of Equation (3), and the squares depict the numerical estimation of *P*_*C*_. Note that according to Equations (1) and (2), τ is measured in units of the neuronal rate dynamics, which are typically in the range of about 10 ms. In the absence of noise (blue line), the readout mechanism discriminates correctly, *P*_*C*_ = 1, for any positive τ. As the noise level increases, the probability of a correct discrimination deteriorates for any given τ, and higher values of τ are required to obtain the same level of accuracy. Thus, the neural noise sets the scale of the sensitivity that this readout mechanism can achieve. In other words, for larger noise levels the signal, τ, has to be scaled up to achieve the same level of performance; Figure 3 inset. Note that the accuracy of the tWTA readout has already been addressed in the past, (Shamir, 2009; Zohar et al., 2011; Shriki et al., 2012). Here we focus on the dynamical system that can implement this computation. However, the parameter σ does not reflect noise in the input that limits the information in it, but rather an inherent variability of the readout mechanism itself that limits its sensitivity.

Rate models describe neural activity by a continuous parameter, and as a result can react immediately to changes in their input. Real spiking neurons have an inherent delay before emitting a spike, and even then their response is sparse in time. This makes it crucial to test the generalization of our results beyond the threshold-linear rate model to a model of spiking neurons. For this purpose, we simulated a tWTA readout mechanism based on competition by reciprocal inhibition between two populations of *N* Hodgkin-Huxley neurons. This was facilitated by the mapping between rate and conductance-based models (Shriki et al., 2003). The dynamic equation for the membrane potential of neuron *i* in population *a* ∈ {1, 2} is as follows

$$\begin{array}{l} C\frac{dV_{a,i}}{dt}=g_{L}\left(E_{L}-V_{a,i}\left(t\right)\right)-I_{a,i}^{active}+I_{a,i}^{ext}+I_{a,i}^{net} \end{array}$$

where *g*_*L*_ and *E*_*L*_ are the leak conductance and reversal potential, *C* is the membrane capacitance, and $I_{a,i}^{active}$ is the voltage dependent current $I_{a,i}^{active}=I_{Na}+I_{K}+I_{A}$. The term $I_{a,i}^{net}$ denotes the reciprocal inhibition from the competing population. Unless otherwise stated, the parameters of the model follow (Shriki et al., 2003); see Methods. The term $I_{a,i}^{ext}$ denotes the input to cell *i* in population *a* ∈ {1, 2} from the input layer that is latency-tuned to the stimulus. In our simulations, $I_{a,i}^{ext}$ is the input current from the upstream population to neuron *i* in population *a*, and is modeled by $I_{a,i}^{ext}\left(t\right)=r\Theta \left(t-\left[T+\tau_{a}+\delta_{a,i}\right]\right)+\xi_{a,i}\left(t\right)$, where *r* is the strength of the response of the upstream population to the stimulus, ξ_*a, i*_(*t*) is a stimulus-independent white noise of the upstream population, *T* and τ_*a*_ are the absolute and stimulus selective delays, respectively, and δ_*a, i*_ represents the trial-to-trial variability in the delay of the input to the neuron. We modeled {δ_*a, i*_} by i.i.d. Gaussian random variables with zero mean and a standard deviation Δ. The term *I*^*net*^ denotes currents resulting from the lateral connection. Specifically, in this case *I*^*net*^ is the reciprocal inhibition, and its strength is governed by the synaptic strength *g*_*s*_ (see Methods for more details).

As we are interested in a WTA-type competitive readout mechanism, the reciprocal inhibition must be sufficiently strong to enable a bi-stable regime in which one population can outrival the other and fully suppress its activity. For this reason, we need to set the total maximum conductance of population internetwork (inhibitory) connection to a level that causes the network to be in a winner-take-all state (just over 0.7μF/cm^2^).

**Figure 4:**
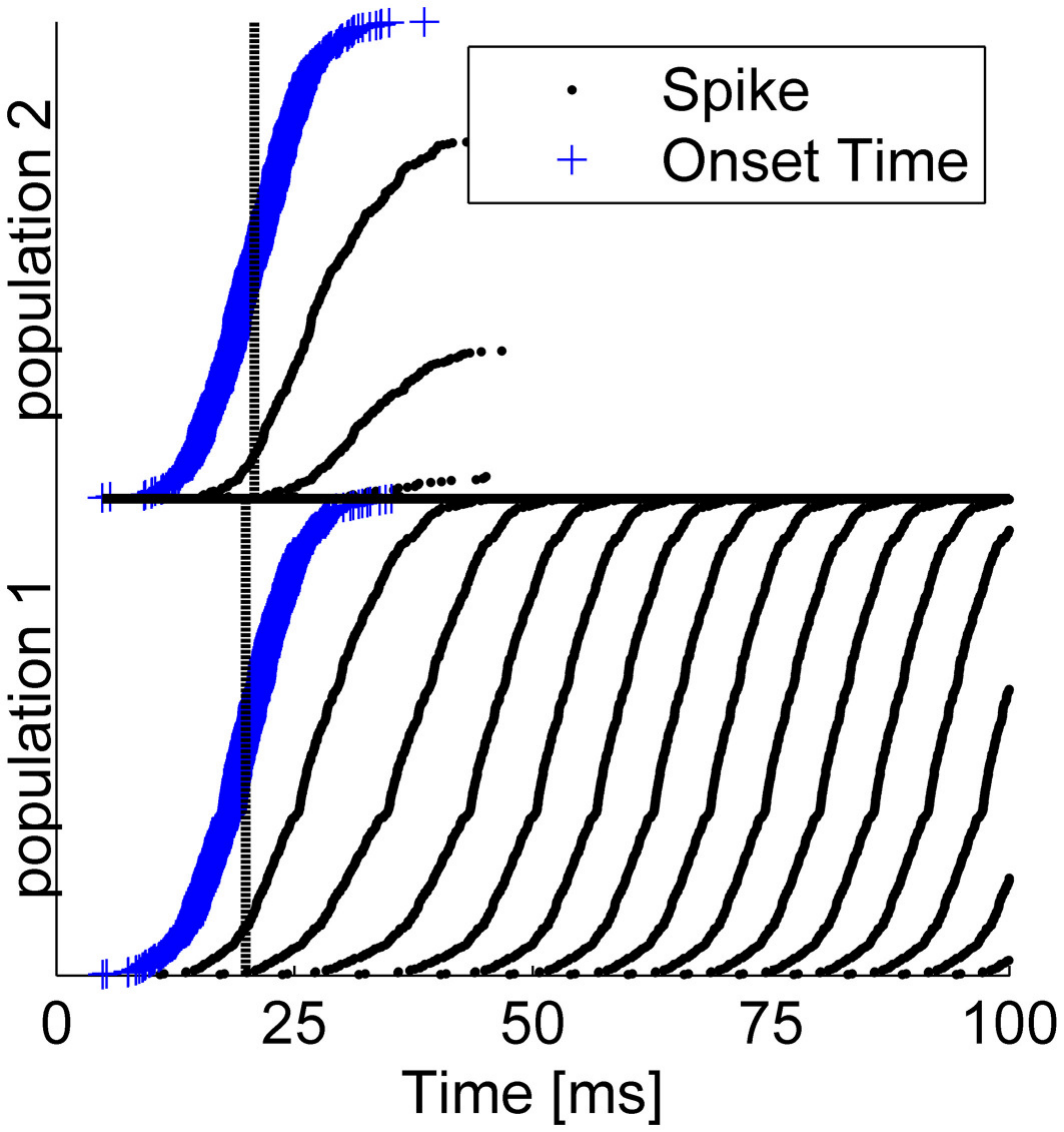
**Typical example of tWTA competition between two populations of 1000 neurons during a single trial**. The Figure depicts a “population raster” where each row shows the spike times of a single neuron (black dots). Bottom half shows neurons from population 1, and upper half population 2. The blue “+” signs depict the specific input latency time for each neuron (*T* + τ_*a*_ + δ_*a, i*_), and the neurons within each group are arranged according to their input latency. The dashed vertical line shows the mean latency for each group, *T* + τ_*a*_. Here we used *T* = 20ms, τ_1_ = 0ms, τ_2_ = 1ms,Δ = 5ms, and $g_{L}=0.71{\text{mS}/\text{cm}}^{2}$.

Figure 4 shows the result of a competition between the two populations of the readout mechanism during a single trial with: *T* = 20ms, τ_1_ = 0ms, τ_2_ = 1ms (*T* + τ shown by the dashed lines), and Δ = 5ms. The blue plus signs (“+”) denote the specific onset time of the input to each cell within this trial, *T* + τ_*a*_ + δ_*a, i*_. The neurons in each group are arranged in the Figure according the latency of their input (early to late from bottom to top). Note that the mean latency difference between the inputs to the two populations was τ ≡ τ_2_ − τ_1_ = 1ms, whereas within each group input latency varied with a much greater standard deviation of 5 ms. Given the considerable variability in the input times to single cells, some neurons in population 2 fired up to three spikes in response to the stimulus. Nevertheless, within several tens of milliseconds the reciprocal inhibition dynamics converged to a state in which population 1 fired with a high rate and the activity of population 2 was fully suppressed (Figure 4). Thus, even though in their steady state both populations received the same input level, this relatively small mean latency difference was sufficient to enable the reciprocal inhibition mechanism to “choose” the correct group in this specific example.

To evaluate the extent to which the result in Figure 4 is typical, we estimated numerically the probability of correct discrimination. To do so, we first needed to define an objective criterion for a “correct” response by the system. In our simulations we chose the winner in the WTA competition to be the population that fired more spikes during the time interval of 50ms ≤ *t* ≤ 100ms. Other choices yielded qualitatively similar results.

**Figure 5:**
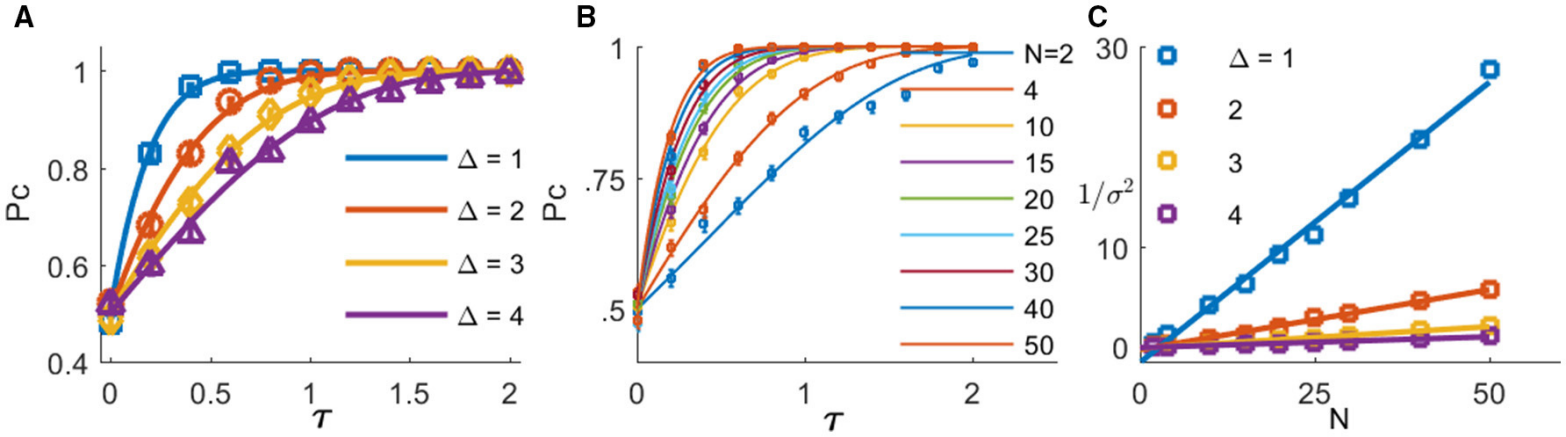
**The probability of correct response of the tWTA is plotted as a function of the stimulus selective delay,τ, (A) for different levels of noise in the input latency, Δ(different colors, Δ is measured in ms, input population size of *N* = 50 was used) (B) for different sizes of input populations (different colors, Δ = 1 ms was used)**. The open markers show the numerical estimation of the accuracy of the conductance-based tWTA competition between two populations of *N* neurons, and the error bars show the SEM. The accuracy was estimated by averaging over 1000 simulated trials. The tWTA decision was the preferred stimulus of the population that fired the most spikes during the time period of 50–100 ms following stimulus onset at *t* = 0. The solid lines show the analytical approximation of Equation (3) using *J* = *2* and fitting σ, with time rescaled by the membrane time constant τ_*m*_ = *g*_*L*_/*C*. **(C)** The scaling of the internal noise with the size of the input population. The (inverse of the square of the) fitted parameter σ is shown as a function of the input population size for different values of Δ (open symbols in different colors, Δ is measured in ms). The solid lines show linear approximations, for comparison. In the simulations we used *T* = 20ms, τ_1_ = 0ms, τ_2_ = τ, and $g_{L}=0.71{\text{mS}/\text{cm}}^{2}$.

Figure 5A shows the probability of the Hodgkin-Huxley type model to discriminate correctly between the two alternatives as function of the mean input latency difference between the two populations, τ ≡ τ_2_ − τ_1_, for different levels of within-group onset variation Δ (shown by the different colors). The solid lines are the analytical approximation of Equation (3) with *J* = 2 and fitted values of σ (note that time in Equation (3) is measured in units of τ_*m*_ = *g*_*L*_/*C*). As can be seen from the figure, even small mean latency differences of less than τ≈ 1 ms can be detected with a high reliability by this simple mechanism. Note, that it is expected that in the limit of large input population sizes the results of the rate model will hold, and in particular Equation (3). Figure 5B shows the probability of the spiking neurons model to discriminate correctly between the two alternatives as function of the mean input latency difference, τ ≡ τ_2_ − τ_1_, for different sizes of input populations (shown by different colors). As can be seen from the figure, even though discrimination accuracy deteriorates with the decrease in population size, this simple readout mechanism is still able to extract information from the neuronal response latencies. Furthermore, fitting the analytical approximation of Equation (3) (solid lines), we find that the parameter that reflects the noise in the input population in the rate model, σ, decays to zero as one over the square root of the input population size, Figure 5C. Hence, one may attribute the deterioration in discrimination accuracy to a decrease in signal-to-noise ratio in the responses of the input populations for smaller population sizes.

## Discussion

Previous studies of the WTA algorithm have focused on the conventional rate-WTA decision mechanism. However, a number of studies reported that the WTA is also sensitive to the temporal structure of its inputs (Coultrip et al., 1992; Lee et al., 1999; Jin and Seung, 2002; Sheliga et al., 2006; Standage et al., 2005; Kurt et al., 2008). Here we focused on the ability of a dynamical system to implement a specific latency-based decision mechanism; namely, the tWTA readout. We found that the simple reciprocal inhibition architecture of the WTA mechanism is sufficient to implement the tWTA. To implement the tWTA we assumed that the system was in the “strong inhibition” regime (*J* > 1), such that it was bi-stable when the inputs to the two competing population were similar. This condition was needed, as we assumed that the input strength to both populations was similar and that response latency alone depended on the stimulus. However, in many cases neurons tend to respond with a higher firing rate to the same stimuli to which they respond with a shorter latency (Zohar et al., 2011; Shriki et al., 2012). In these cases the assumption of “strong inhibition” can be relaxed.

The robustness of the reciprocal inhibition mechanism for latency coding was tested against noise in the initial conditions. Additional parameters that govern the tWTA dynamics may also vary. In particular, we assumed that the reciprocal inhibition between the two populations is identical. This assumption contributed to the symmetry that is underlying the infinite sensitivity of the tWTA mechanism in the absence of noise. How will asymmetry in the reciprocal inhibition affect the tWTA? Assume, for example that the effective inhibition from population 2 to 1, *J*_1←2_, is stronger than from population 1 to 2, *J*_2←1_. We find that as long the system is in the strong inhibition regime, i.e., *J*_2←1_, *J*_1←2_ > 1, then for sufficiently large τ the system will converge to the correct fixed point (in the absence of noise). However, for any positive difference, (*J*_2←1_ − *J*_1←2_) > 0, there exists a critical value τ_*c*_, such that for any τ < τ_*c*_ (here positive τ denotes input to population 1 preceding input to population 2) population 2 will win the tWTA competition. The critical value τ_*c*_ decays to zero as the system approaches symmetry, (*J*_2←1_ − *J*_1←2_) → 0. Consequently, an additional “learning mechanism” that can fine-tune the reciprocal inhibition is required if high sensitivity for very short latencies is necessary. Note that the parameter *J* reflects the effective inhibition strength that is a product of the strength of a single synapse by the number of synapses.

**Figure 6:**
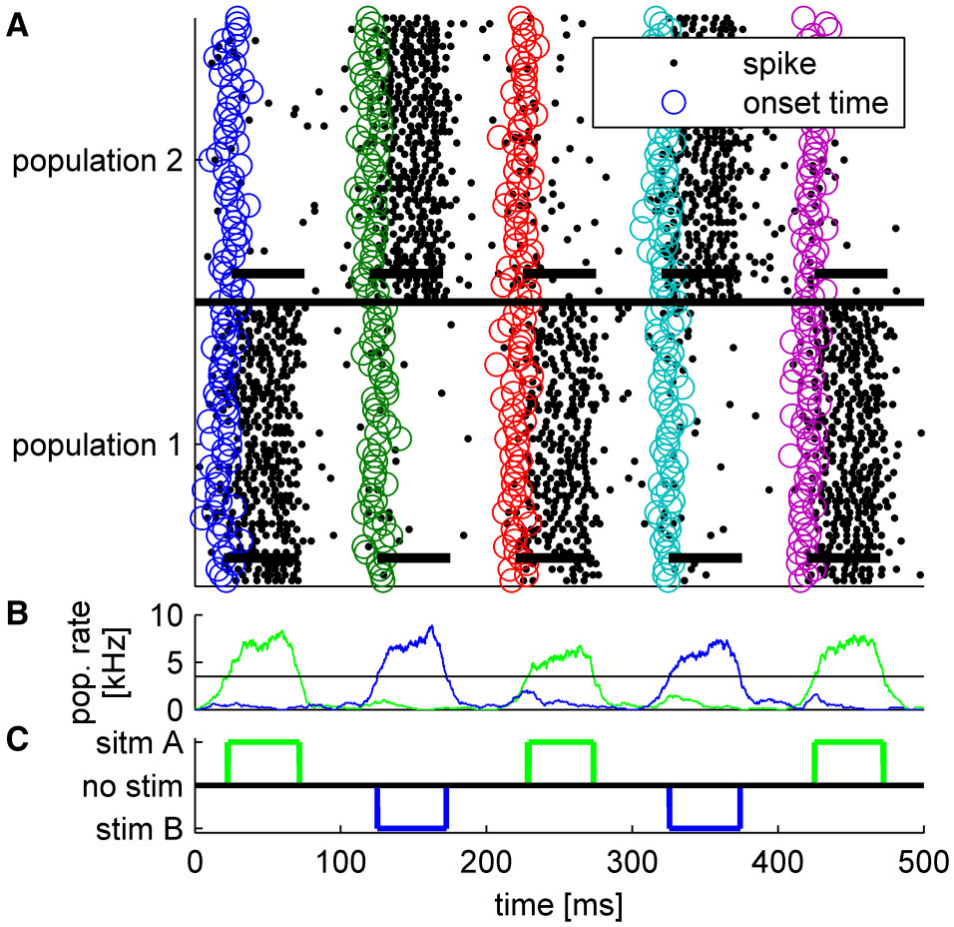
**Typical example of tWTA competition between two populations of 50 neurons during a single trial in which a series of five alternating stimuli were presented every 100 ms**. Thus, for the first presentation *T* = 20 ms, for the second *T* = 120 ms, and so on. Each stimulus lasted Δ*T* = 50ms. **(A)** Population raster plot: each row shows the spike times of a single neuron (black dots). Bottom half shows neurons from population 1, and upper half population 2. The open circles depict the specific input latency times for each neuron (*T* + τ_*a*_ + δ_*a, i*_) in different colors for the different presentations. The thick horizontal lines show the mean times in which each population received input; i.e., from time *T* + τ_*a*_ to time *T* + Δ*T* + τ_*a*_. **(B)** The population firing rate for population 1 (green) and population 2 (blue). The rate was estimated by the total spike count in a sliding window of a 10 ms time bin. Horizontal black line shows the decision threshold of 3.5 spikes/ms used to generate C. **(C)** The decision of the tWTA network. In the simulations we used τ = 0ms for the preferred stimulus, τ = 5ms to the un-preferred, and $g_{L}=1.1{\text{mS}/\text{cm}}^{2}$.

**Figure 7:**
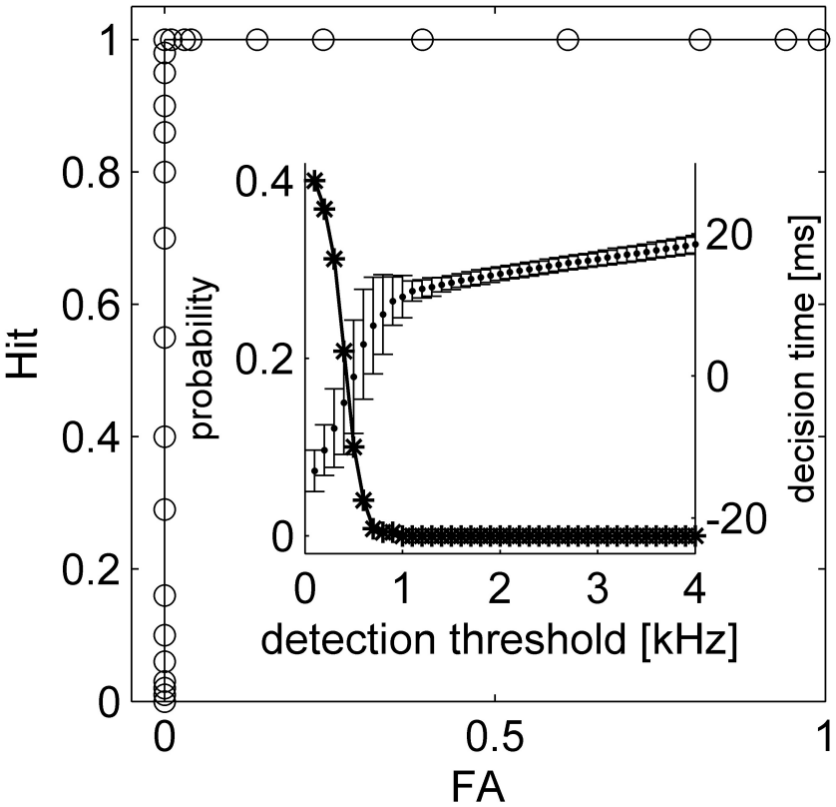
**The tWTA onset detection accuracy**. The stimulus detection ROC is presented, depicting hit probability (correct detection) as function of false alarm. Here, onset detection corresponds to a “detection” of a stimulus during the absolute delay period, 0 < *t* < *T*. Consequently, false alarm may scale with the absolute delay period, *T*. Moreover, a false alarm may be flagged in the absence of stimuli due to spontaneous firing of the neurons. Thus, a more relevant characterization of false alarm for stimulus onset detection task would be the false alarm rate during spontaneous activity. The inset shows average decision time and STD for different criterions (dots and bars, respectively) and the probability of having at least one false alarm during 1 s of spontaneous activity as a function of the detection threshold (asterisk). The parameters used for the simulation are the same as the parameters in Figure 6.

It has been argued that latency code readouts require an additional stimulus onset detector which serves as a reference from which latency can be measured. A number of studies have reported that onset can be detected by pooling information from neurons that show poor latency tuning (Zohar et al., 2011; Brasselet et al., 2012; Shriki et al., 2012). Here we suggest that such an additional mechanism is not essential. Figure 6A shows the spiking response of the tWTA network to a series of stimuli. In the first, third and fifth presentations the mean latency of the inputs to population 1 was shorter, whereas in the second and fourth presentations population 2 received the fastest input (on average). By examining the population firing rate in Figure 6B, stimulus1, stimulus 2, and no stimulus can easily be discriminated, by adding a decision threshold (black horizontal line in Figure 6B). Setting a reasonable value for the decision threshold results in correct detection and discrimination for all 5 presentations without the use of an additional neural population or detectors; Figure 6C. Figure 7 quantifies the tradeoff between generating false alarms at a high rate by setting the decision threshold too low, and failing to detect the stimulus by setting the threshold too high. The considerable difference between the spontaneous firing rate and the firing rate in response to the stimulus allows for a high detection probability and a negligible false alarm rate, as shown by the sharp ROC curve. However, decision threshold also affects the speed of the response and the probability of false alarm during spontaneous activity; Figure 7 inset.

**Figure 8:**
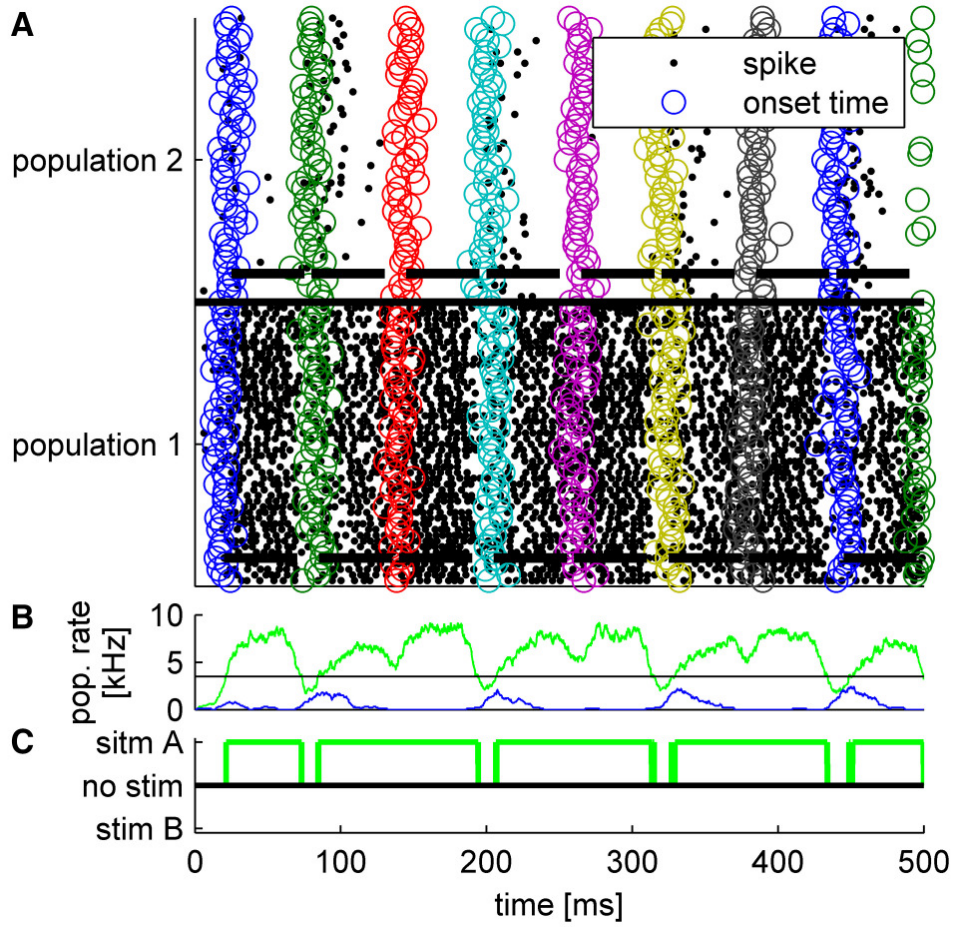
**Typical example of tWTA competition between two populations of 50 neurons during a single trial in which a series of five alternating stimuli were presented every 60 ms**. Thus, for the first presentation *T* = 20 ms, for the second *T* = 80 ms, and so on. Each stimulus lasted Δ*T* = 50ms. During the odd presentations (first, third, fifth…) population 1 received the faster input whereas in the even presentations population 2. **(A)** Population raster plot. Each row shows the spike times of a single neuron (black dots). Bottom half shows neurons from population 1, and upper half population 2. The open circles depict the specific input latency times for each neuron (*T* + τ_*a*_ + δ_*a, i*_) in different colors for the different presentations. The thick horizontal lines show the mean times in which each population received input; i.e., from time *T* + τ_*a*_ to time *T* + Δ*T* + τ_*a*_. **(B)** The population firing rate for population 1 (green) and population 2 (blue). The rate was estimated by the total spike count in a sliding window of a 10 ms time bin. Horizontal black line shows the decision threshold of 3.5 spikes/ms used to generate C. (**C)** The decision of the tWTA network. In this simulations we used τ = 0ms for the preferred stimulus, τ = 5ms to the non-preferred stimulus, and $g_{L}=1.1{\text{mS}/\text{cm}}^{2}$.

The tWTA mechanism does not operate like a working memory but rather more like a sensory system. Thus, in the example in Figure 6 the neural activity decays to a spontaneous firing rate after each stimulus offset. However, this decay is not instantaneous. Therefore, if the inter-stimulus-interval is very brief, the tWTA decision may be affected by its past. This scenario is illustrated in Figure 8, in which due to the short inter-stimulus-interval the tWTA decision is dictated by the preceding decision rather than by the stimulus itself. This is a hallmark of tWTA competition which may serve as an empirical prediction both on the psychophysical as well as the neural level. In this respect the well-studied psycho-acoustical phenomenon termed the *precedence effect* (Litovsky et al., 1999) may have some bearing. In the precedence effect, when estimating sound source location, the perception of the delayed stimulus is suppressed. This is believed to assist in overcoming the corrupting effects of echoes in the computation of the sound source location.

Conventional rate-WTA mechanisms have been suggested to play an important role in various computations in the brain and in numerous systems (Lee et al., 1999; Standage et al., 2005; Sheliga et al., 2006, 2007; Kurt et al., 2008). Their implementation only requires a very rudimentary reciprocal inhibition design. Nevertheless, even this simple architecture yields a mechanism that is intrinsically sensitive to the response latency of its inputs. Consequently, by construction, almost every conventional WTA mechanism is highly sensitive to latency cues as well. This highlights the possible role of latency as a source of information in the central nervous system.

## Author contributions

OZ, MS: Methodological, analysis, and interpretation of results.

## Funding

This work was supported in part by Israel Science Foundation ISF grants No 722/10 and 300/16, and by the Helmsley Charitable Trust through the Agricultural, Biological and Cognitive Robotics Initiative of Ben-Gurion University of the Negev.

### Conflict of interest statement

The authors declare that the research was conducted in the absence of any commercial or financial relationships that could be construed as a potential conflict of interest.
